# Adolescent motherhood: reflections based on a clinical case

**DOI:** 10.1192/j.eurpsy.2025.1105

**Published:** 2025-08-26

**Authors:** H. Ben Mustapha

**Affiliations:** 1 Essonne, Barthélemy Durand Public Health Establishment, Île-de-France, France

## Abstract

**Introduction:**

Adolescence and motherhood are transitional phases, moving from childhood to adulthood and from womanhood to motherhood. These processes involve significant psychological conflicts, where childhood narcissistic vulnerabilities risk being exposed.

**Objectives:**

The study aims to describe the mother-infant bond and the management of adolescent motherhood. This case informs the psychopathological and therapeutic reflections throughout the study.

**Methods:**

This study examines a clinical vignette of a 16-year-old adolescent followed in the Perinatal Department at Rouen University Hospital, alongside a literature review. Semi-structured interviews were conducted with the adolescent mother and her family from July 2019 to April 2020, covering sociodemographic data, pregnancy experiences and consequences, the adolescent’s baby, the baby’s father, and the adolescent’s family. Written consent was obtained from the mother at the study’s outset.

**Results:**

The case involves M.D, a 16-year-old at 16 weeks gestation, is the youngest of two siblings and is in a relationship with a 25-year-old man. Her family history includes maternal depression, and her personal history includes ADHD with irregular follow-up, behavioral hospitalization at age 7, and a pregnancy termination in 2018. She was referred by a midwife due to challenges in envisioning her pregnancy. M.D. lives in a disrupted family dynamic marked by intrafamilial violence, conflicts with her brother, and repeated runaways. She was placed under social services following a report from her mother concerning behavioral issues. Her pregnancy was marked by anxiety over a potential forced termination, conflicts between adolescence and motherhood, and worries about childbirth. Motherhood posed additional challenges, such as infantile regression, irritability, impulsivity, difficulty caring for the baby, emotional immaturity, and ambivalence toward her pregnancy and motherhood. Her interactions and emotional attunement with the baby were inadequate. M.D. received multidisciplinary support (gynecologist, pediatrician, social worker, psychiatrist, child psychiatrist, psychologist, childcare provider) with extensive family and partner involvement. Improvements were noted in mother-infant bonding, reduced impulsivity, and decreased marital conflicts with Methylphenidate.

**Conclusions:**

A collaborative care network is essential to support the bond between the adolescent mother and her child. The father’s role can serve as a separating third party, facilitating the child’s access to symbolic thinking. This role may also be filled temporarily by professionals who act as substitute third parties, supporting both the mother’s and child’s development.

**Disclosure of Interest:**

None Declared

